# Co-design with Integrated Care Teams: Establishing Information Needs

**DOI:** 10.5334/ijic.7618

**Published:** 2023-10-19

**Authors:** P.J. White, Brian P. Casey, Olga Cleary, Emer Finn, Kate O’Connor, Neville Coen

**Affiliations:** 1 Humanities, South East Technological University, Carlow, Ireland; 2 HSE Research and Development, HSE. Contact details: 2^nd^Floor Jervis House, Jervis Street, Dublin 1, Ireland; 3 Memory Technology Resource Room (MTRR) New Ross Healthy Living Centre, New Ross, Wexford Ireland, Y34 C821, Ireland; 4 Carlow, Kilkenny and South Tipperary, James Green Community Services, Kilkenny, Ireland; 5 Waterford Integrated Care for Older People (WICOP), Health Service Executive, St Patrick’s Way, Waterford, X91 XE86, Ireland

**Keywords:** integrated care, co-design, information needs, information, communication, integrated working

## Abstract

**Introduction:**

Co-design has been cited as playing a major role in the future of effective integrated care, however, there is a lack of reporting and reflection on the methods used. Information sharing is fundamental when working in integrated care, however sharing across professions, service settings and localities can be complex. Through co-design, we seek to establish a shared understanding of information needs within a newly formed integrated care team. In doing so we aim to inform future practice in the understanding of co-design.

**Description:**

Co-design Workshop 1 (N = 24 participants, plus 6 facilitators), collected ‘Current Position’ understanding of service information needs. Co-design Workshop 2 (N = 18 participants, plus 6 facilitators) sought a ‘Future Position’ understanding, identifying solutions and next steps for establishing information-need solutions. Reflection on the co-design process was conducted to inform future co-design practices.

**Conclusion:**

Identified was a wide range of future service information needs under the themes of Culture Building, Health System Needs, and Processes. We conclude with 4 key learning points on co-designing. 1. Ensure simplicity in format. 2. Interdisciplinary co-design and co-facilitation of workshops are beneficial. 3. Planning and preparation are key. 4. Co-designing can enhance communication for service improvement.

## Introduction

Information sharing is fundamental when working in integrated care, however sharing across professions, service settings and localities can be complex. Co-design methods have been cited as playing a major role in the future of effective integrated care, however, there is a lack of reporting and reflection on the methods used. To support a newly formed community integrated care team in their shared goal of integrated care, we seek to establish a shared understanding of information needs through co-design. To inform future methods and practice, we report and reflect on the process. In doing so we aim to inform future practice in the understanding of co-design.

### Background Context

The ‘Sláintecare’ programme was established in 2017 to transform Irish health and social care services and to create a universal single-tier system [1]. Sláintecare’s quadruple aim is to improve patient/service user experience, improve clinician experience, lower costs and achieve better outcomes [2]. In achieving these aims, there is a need to re-orientate the model of care towards integrated and community care [2]. Improving clinician experience in integrated and community care requires a deep understanding of stakeholder needs for effective service change to optimise healthcare system performance and enhance patient/service user experience and outcomes.

Published in 2014, the ‘*Community Healthcare Organisations – Report and Recommendations of the Integrated Service Area Review Group*’ sets out a blueprint for how the Health Service Executive (HSE) primary and social care services will be organised and managed in the community [3]. Community Healthcare Networks (CHNs) are a foundational step in building a better health service.

Community care structures are now established, including 96 Community Healthcare Networks (CHN) aligned to 9 Community Healthcare Organisations. Each CHN operates across an average population of 50,000 with between 4–6 multi-disciplinary care teams working together to deliver the Sláintecare vision of providing the right care, in the right place at the right time. CHNs will enable healthcare decisions to be made closer to the point of care, and specific to population needs with an emphasis on coordination and integration between community services and between community and acute service [4,5,6]. The goal is also to develop strong relationships with local communities and tailor service delivery to suit the needs of the population.

An integral part of integrated care is that professionals from different disciplines and services work together to provide high-quality care, this requires a high degree of information exchange about and with patients [7]. As CHNs are established, a key challenge is developing mechanisms to support information-driven healthcare within the limitations of clinical and health and social care information systems.

### The Need

Team engagement and information sharing are fundamental aspects of integrated care working. Major efforts have been made to move healthcare services from silos of institutional-type care into community settings. However, despite progress services remain fragmented and in many cases, access to information is limited with data systems and infrastructure underdeveloped and team working weak [8].

In the Irish health service, Health and Social Care Professionals (HSCPs) are the second largest clinical group representing 25% of the clinical workforce and 14% of the overall health service workforce [9]. To promote integrated working, HSCPs may need to change the way they work, strengthen the delivery of ‘end-to-end’ care and enable the closing of linkages between primary and secondary care [10]. For HSCPs to promote integrated working and to improve service user outcomes, effective and appropriate communication is a specific competency [11]. There is a need then for HSCPs to have relevant filtered, focused and accessible information available to support effective patient care directly and across their team. Efficient information sharing has the potential to mitigate adverse impacts of excessive administrative burden on clinicians in favour of clinical care [12]. Several initiatives are underway to address these challenges in community healthcare nationally, which will require translation into more local integrated care contexts. Establishing an understanding of HSCPs information needs for integrated care work is required in the first instance.

### Benefits and Need for Co-Design in Healthcare and Integrated Care

Integrated care is adaptive to suit the needs of the service users, therefore there is a need to understand specific local contexts, values and preferences [13]. As we prepare for structural change in our health services, HSCPs responsible for the delivery of community healthcare services in CHNs must contribute to shaping the future of care in our community. The most successful international integrated care programmes have adopted a bottom-up person-centred approach involving service users and clinicians [14]. Co-design has been identified by the HSE as a crucial methodology to achieve this, with a key reform theme being to: “…work in partnership with service users, to co-design new ways of improving the experience of care, and support staff in the delivery of services” [15].

Co-design is now used as a key method in healthcare reform. According to Ní Shé & Harrison, co-design in healthcare is the process of bringing together service users, clinical and non-clinical staff, and relevant groups to work together to improve services [16]. It involves the equal partnership of individuals who work within the system and individuals who have lived experience of using it [17]. Co-design is intended to be meaningful engagement; occurring across all stages (of project work), and it can range in intensity from passive to high involvement [18].

When discussing co-design, it is important to note definitions from a design practitioner’s viewpoint. For example, Sanders and Stappers describe co-design as the “creativity of designers and people not trained in design working together in the design development process” [19] This definition points to the important role of trained designers to inform the process and ensure that designing is central to the process.

According to Moll et al., the co-design process seeks to “tap into tacit knowledge, creativity and shared meaning of diverse perspectives to co-create a shared vision for improvement” [20].

The distinguishing feature of co-design processes led by designers is the involvement of forms of ‘making’ in a practice based manner e.g. prototyping. These allow participants to explore, share and test a vision of improvement with others [21]. Roberts et al, states that “…the application of design approaches within healthcare help drive necessary innovation in care delivery models [22] p. 4. Furthermore, leveraging the knowledge, experiences, and insights of end-users is proven to achieve impactful innovations, benefiting researchers, practitioners, processes, and research outcomes [18,23]. Wolstenholme, Grindell and Dearden state that co-design can lead to healthcare teams exhibiting characteristics that support innovation in the long term [24]. When used to improve healthcare systems, Langley, Wolstenholme and Cooke, report that it creates the right conditions for knowledge to be mobilised and activated [21], helping to make sure that improvements are useful and relevant [23]. Co-designing is advantageous when working with teams as it leads to more long-term success, more support and enthusiasm for change, and can generate solutions that improve day-to-day experiences [25,26].

Co-design methods and processes have been cited as playing a major role in the future of effective integrated care. However, a knowledge gap lies in the underdevelopment of co-design processes in health care and HSCPs involvement in this. This knowledge gap is a result of a lack of reporting and reflection on using methods in the field. Ward et al. state that although co-design has great potential as a methodology for understanding healthcare; there is a gap in the peer-reviewed literature on ‘how to do’ co-design in practice [17]. Noting the need for a co-design approach in healthcare, Wolstenholme, Grindell and Dearden, state that further exploration of the use of design in service improvement is required [24]. At a national level, there is a need for HSCPs to co-design the planning of services across all aspects of health service delivery [9].

### Aims and Objectives

Due to the needs and knowledge gap outlined above, the aim of this paper is to inform future practice in the understanding of the development, use and facilitation of co-design with integrated care teams.

The specific objectives of the paper are to:

1. Report on co-design development
  Report on the development, use, and facilitation of co-design methods with an integrated care team. In doing this we seek to establish a shared understanding of integrated care service information needs to support good practice and quality improvement at Community Health Network level.
2. Reflect on the experience of co-designing.
  Offer a reflection on co-designing used within integrated care to inform the ‘how to do’ of future practice. In this paper, we reflect on the use of co-design within a newly formed integrated care team seeking to improve health service provision in the community.

## Methodology

**Figure 1:**
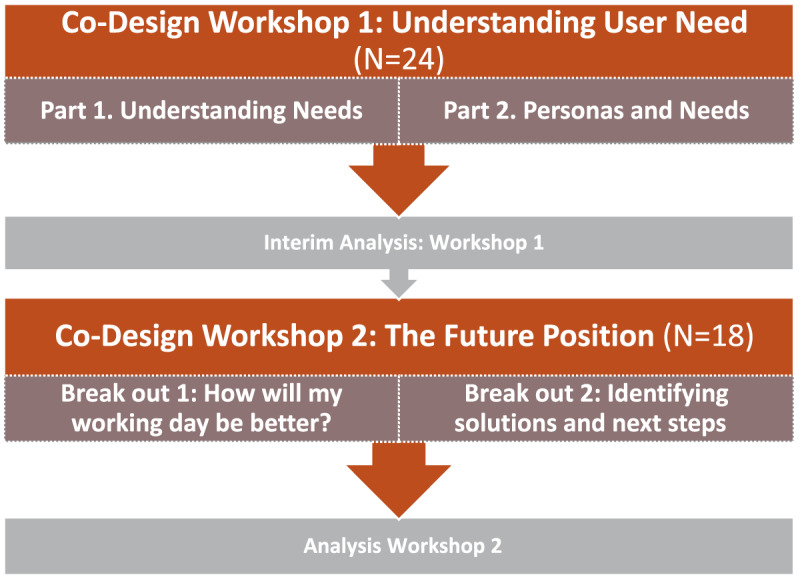
Co-design stages.

To meet the aim and objectives, co-design methods were developed and used over 2 co-design workshops to support service understanding of information needs for clinicians, HSCPs and health service management operational in a newly formed Community Healthcare Network. The identified participants were interdisciplinary, consisting of Occupational Therapy, Physiotherapy, Speech and Language and Dietetics as well as engagement with Public Health Nursing, General Practice, Health and Well-being and Primary Care Management (Workshop 1: N = 24 and Workshop 2: N = 18). The facilitation team (N = 6) was also interdisciplinary, consisting of two experienced co-design facilitators from Product Design and Design Engineering disciplines, two HSCPs and two healthcare managers, including the CHN manager. The facilitation team designed, planned and prepared the structure and original content for the workshops purposefully to tailor directly to the needs of the HSCPs. The co-design workshops were conducted and facilitated in person in keeping with COVID-19 guidelines. Overview of Co-design stages are shown in Figure 1.

For co-designing, a design-led qualitative interpretivist approach was taken to engage with stakeholders and a grounded theory approach to analysis. Interpretivist approaches were used to ensure shared understandings and meanings with participants [27]. Due to the inclusive nature of its process [28], participant-led co-design through a practice-based approach was the core methodology used. This ensured that stakeholders designed from not only their point of view but also shared and drew from the experiences of others, guaranteeing that a deep, empathic, and unbiased understanding is gained.

Reflections on the experiences of co-designing were conducted by and were from the design and facilitation team using the Gibbs reflective cycle. In this, the facilitation team engaged in collaborative reflection sessions where they shared their observations and insights. This occurred after each workshop. Describing and evaluating the positive and negative feelings and thoughts of the experience, analysing to make sense of the situation and conclusion about what was learned and what you could have done differently for the future [29].

The HSE National Framework for the Governance, Management and Support of Health Research (RGMS Framework) outlines the necessary guidelines for the proper governance and management of research within healthcare services and applies to all health research hosted by HSE or HSE-funded services. After consulting with the national HSE Research and Development Office, guidance was given that as this was a service quality improvement initiative with a focus on co-design methodology, it was deemed to fall outside the scope of the HSE RGMS Framework. Quality improvement falls under section 4.1.4 Activities that do not require Research Ethics Committee (REC) approval [30].

There was no obligation for participation but those who had an interest in service improvement and the capacity/availability to attend were encouraged, no incentives were offered to participate. Participation was encouraged by the Network Management and supported by senior health services management as part of a service improvement approach. Permission to leave their clinical role was granted for the workshop. Participants weren’t required to complete any pre-work or post-work between events.

Awareness of any potential power dynamics between disciplines and participants was important when facilitating the co-design workshops. This was facilitated by being conscience of equal time and voice of each participant in each group when possible. The design and facilitation team set out the objectives of the workshop in an introductory presentation at both events and highlighted the importance of everyone’s contribution across disciplines and the need to ensure there was a focus on understanding the information needs of all representatives to move towards an integrated team approach in the Network. Participants did not record their names on their ideas during the co-design activities, reinforcing the message that all ideas were equally valued regardless of seniority or discipline. In addition, more junior HSCP representatives as well as discipline managers were purposely recruited to capture diverse information needs thereby ensuring a balanced input from staff grades.

### Limitations

Limitations include the underrepresentation of some HSCPs across workshops as not all HSCPs were represented at both, this was due primarily to the difficulty in obtaining a workshop time and location that worked for all disciplines. All disciplines were requested including Public Health Nurses and Psychology who work within the Network but are not part of the line management process. Psychology could not attend due to staff shortages. A representative sample was present at both workshops, including representation from all disciplines in the CHN. COVID restrictions meant that the sample size was smaller than originally envisaged as workshop attendance had to be maintained at certain numbers to allow for adequate social distancing. Time constraints meant both workshops were limited to a half-day commitment. The focus of the initiative meant that only staff operating within the CHN were included in the workshops and gaps remain in the knowledge base for information needs for integrated working beyond the Network with the wider health system.

Due to funding obligations, project scope, and constraints, the primary focus for these workshops was to establish information needs within integrated care teams e.g., clinical and HSCP staff. Co-design workshops of this nature are a new approach to HSE service improvement, therefore require incremental development, support, and future funding to progress. Acknowledging that they are key to success, future co-design workshops will be planned to include patient and service users. These future workshops will open out divergent questions on information needs ensuring equality and diversity of participant’s voices.

### Sample Participants

The process employed a non-probability purposeful sampling approach, this was appropriate for exploratory enquiry to develop an understanding of the needs of HSE clinicians in the CHN which is a small defined population of clinical and HSCP staff. In a targeted approach, the design and facilitation team (N = 6) used their expertise to draw from the total sample of clinicians to identify a sample most useful to deliver on the outcomes of the study. This is a commonly applied and useful approach in health services sampling.

Sampling criteria included representation from all disciplines in the CHN. It also included representation from management and staff grades to ensure a wide range of experiences and ideas informed the enquiry. The sampling frame is the total number of clinicians and managers in employment in the Network. Recruitment was by email, and the initiative was supported by the Network’s Communications Working Group. Participants were encouraged to attend both events at the outset and again encouraged to attend workshop 2 at the end of workshop 1.

An extended group of participants were considered for inclusion in the study in line with integrated service provision including Public Health Nurses, GPs, practice nursing and pharmacy as well other extended support service providers in the community. There was no obligation for participation but those who had an interest in service improvement and the capacity/availability to attend were welcomed. The final sample of participants was identified across disciplines as outlined in Table 1. We achieved a high representation of the various relevant stakeholders across the sample we were seeking. Clinical staff were a mix of Staff Grade, Senior Grade and HSCP Discipline Management.

**Table 1:** Workshop representation of the two workshops.


DISCIPLINE	WORKSHOP 1	WORKSHOP 2

Occupational Therapy	4	2

Physiotherapy	3	2

Speech and Language	2	2

Dietetics	4	2

Health and Wellbeing	2	2

Public Health Nurse	2	2

General Practitioner Lead	1	1

General Practitioners in the CHN	2	1

HSE Management, Staff and Administration	4	4

Total	24	18


### Co-Design Workshop 1: Understanding User Need

#### Overview

Co-Design Workshop 1 sought to collect current position understanding and highlight gaps in system knowledge and service information needs. The objectives were to:

1. Define parameters of information needs for integrated care working in Community Health Networks
2. Establish a shared understanding of information on current position needs among participants
3. Map existing information flows as ‘current position’ based on patient personas and identify pinch points in system knowledge and integrated information exchange.

The co-design activity was conducted in an active workshop-style setting. A large hotel function area was hired with moveable furniture to allow for flexibility and movement, and large wall spaces to visualise and share ongoing work. This workshop was facilitated in person by the design and facilitation team. Table 2 outlines the agenda for Workshop 1.

**Table 2:** Agenda Workshop 1.


INFORMATION NEEDS FOR INTEGRATED WORKING – WORKSHOP 1 AGENDA	

**8:30**	Tea/coffee, networking

**9:00**	Welcome and introduction

**9:15**	Part 1: Information needs for integrated working – *What do we need to know?*

**10:00**	*Coffee break*

**10:15**	Part 2: Information needs for integrated working – *Identifying the challenges and opportunities*.

**11:00**	Capturing the learning and key themes identified

**11:20**	Wrap up and close


#### Workshop 1 Format

A welcome and introduction presentation formally commenced the workshop. Outlined in the introduction was the importance of the co-design approach advocating that participant input was essential to the development of new ways of working in the Network.

**Figure 2:**
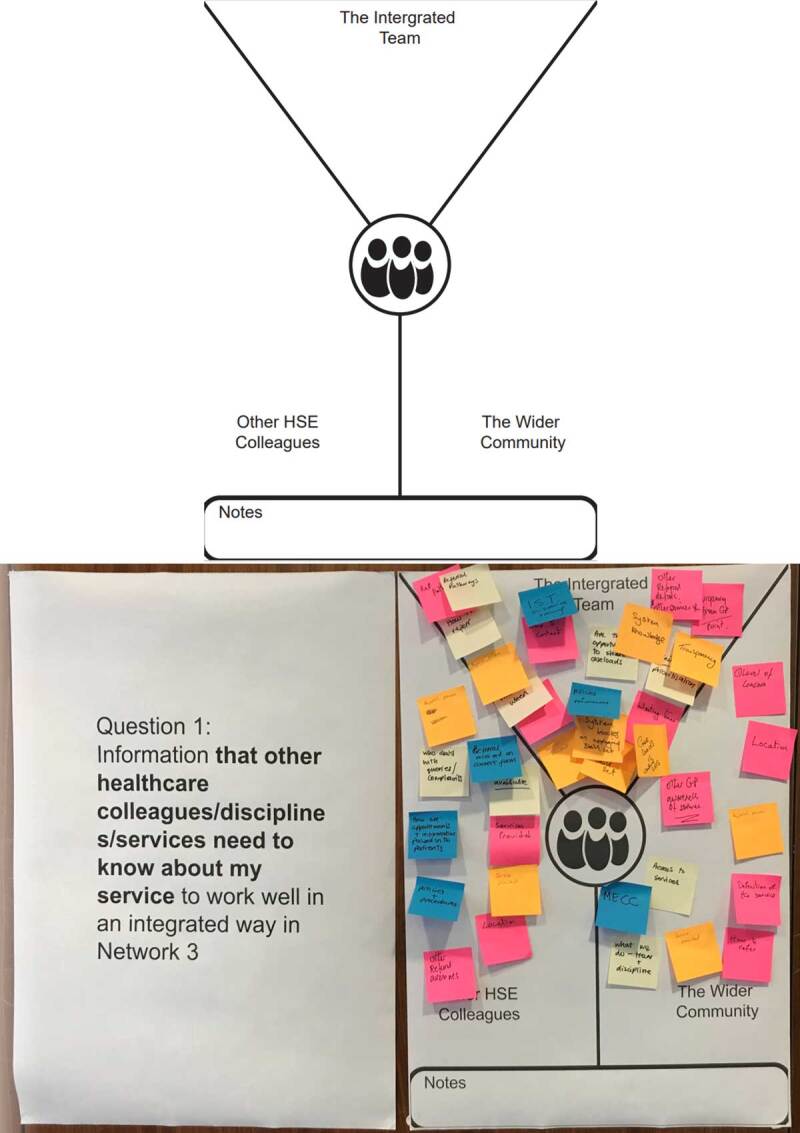
Workshop 1 Part 1 blank ‘Current Position Needs Finding’ poster template and example of poster template in the process of affinity diagramming.

For the workshop, participants were grouped into 6 breakout groups of 4 participants, guided by one of the 6 facilitators. As this work was conducted during the COVID 19 period, services couldn’t release equal staff representatives from each discipline for workshops. Understanding that an equal distribution of disciplines was not possible across all 6 groups, the design and facilitation team selected groups to ensure the optimum diversity of the interdisciplinary range of healthcare providers. The workshop was designed to be participant-led, in doing so being open and discursive [28], collecting as many views, experiences, and narratives of services as possible, and building on and exploring the experiences of others. Through a series of open-ended questions, participants were asked to discuss their experiences of the current position in healthcare services. Poster templates were designed and printed in A1 format to facilitate the capture of experiences (Figure 2). As experiences were shared, they were written on sticky notes and displayed on the posters in a process of affinity diagramming. Affinity diagramming is a method used in User Experience Design to organise large sets of ideas into clusters, commonly used to sort design ideas in group workshop settings [31]. A blank poster template labelled ‘Backlog Items’ was available to each group to display any discussed item that didn’t fit into a theme or issues that were worth noting but could prevent the flow of topic discussion. In the planning stage of the workshop, prototyping of workshop assets and ‘trial runs’ of the process occurred. Iterative revision was implemented to anticipate and mitigate potential adverse responses.

#### Co-design Workshop 1 Part 1

Part 1 of the workshop was guided by the following questions to understand the current position needs:

Initial Warm-up Brainstorm question: What does working well in an integrated way mean to me?

This was divided into the sub-questions:

- What is ‘Working Well’?
- What is Integrated working?
- What are my immediate information needs?

Main Question 1: Information that other healthcare colleagues/disciplines/services need to know about my service to work well in an integrated way in Network 3?

Main Question 2: Information that my service needs to know about other services to work well and deliver integrated care in the Network?

Participants were asked to think about these information needs from three different perspectives:

- As an integrated team in your Network
- With other HSE colleagues
- With the wider community

#### Co-design Workshop 1 Part 2: Personas and Needs

**Figure 3:**
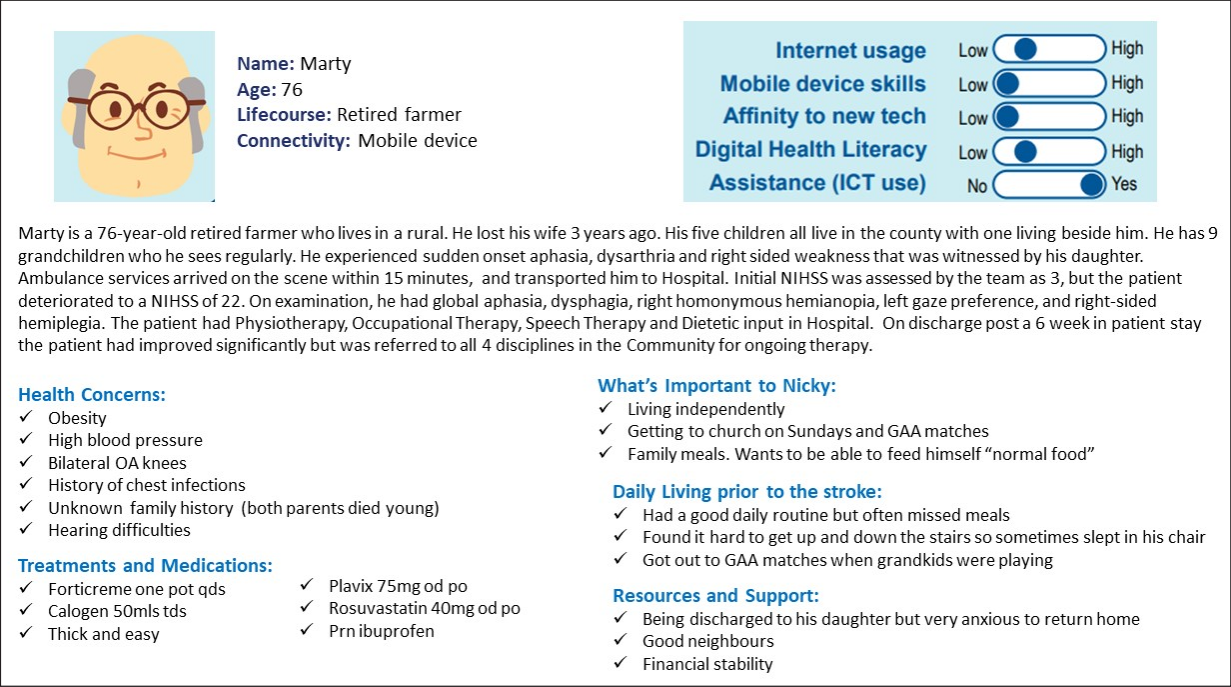
Persona 1. Marty.

**Figure 4:**
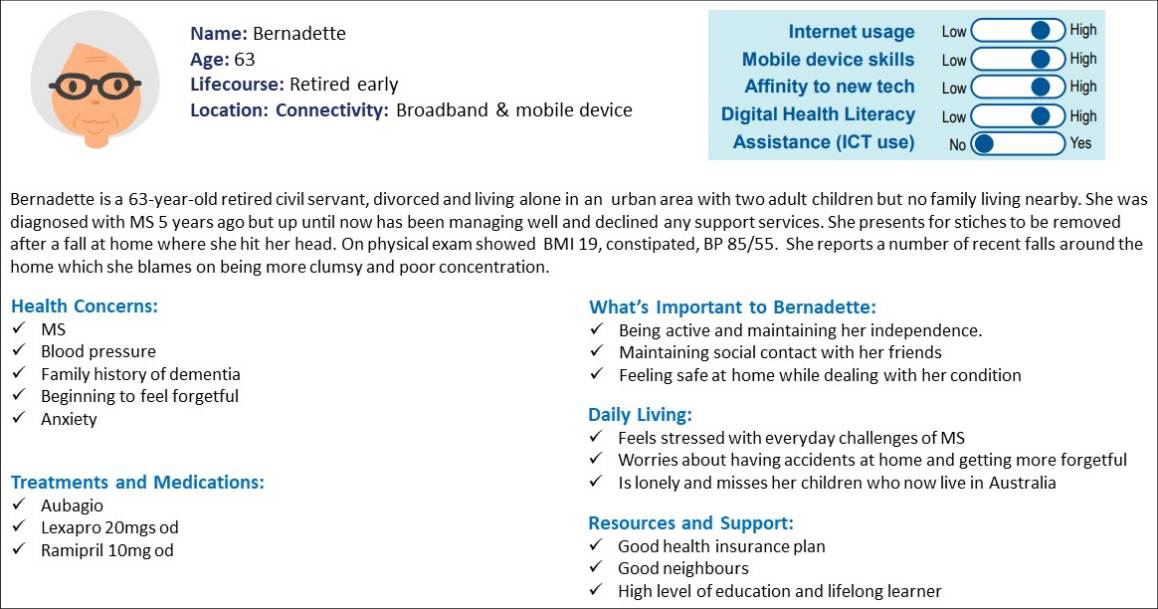
Persona 2. Bernadette.

**Figure 5:**
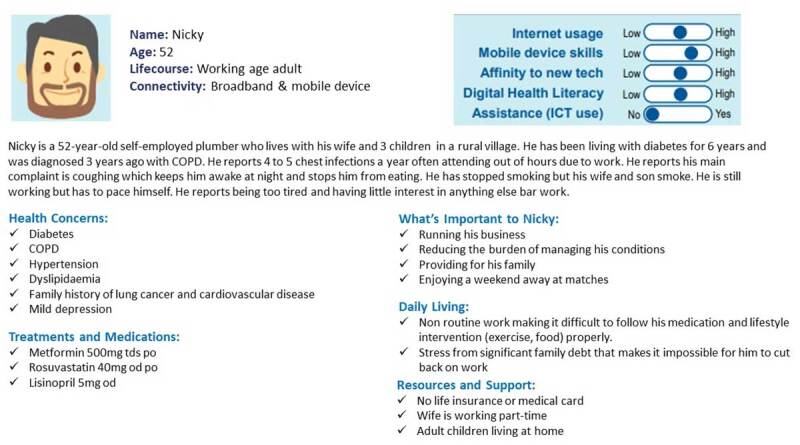
Persona 3. Nicky.

Part 2 of the workshop involved identifying the challenges and opportunities in information needs for integrated working. To facilitate participants, 3 personas with different health and social care needs were created. (Figures 3, 4, 5). These personas were pre-defined and piloted with a sub-group of HSCPs before use in the workshop. Personas are representative “characters” of end-users, made up of multiple people offering a synthesised representation of a population [32].

The following tasks were assigned to the breakout groups:

Task 1: select the persona the group feels they can best discuss

Task 2: individually consider:

- all care requirements for the patient
- all information you need on the patient to deliver the best care possible

Task 3: As a group discuss all the information needs you have to deliver care for the patient in the Network

Task 4: As a group identify any challenges you have to get the right information for this patient

Task 5: As a group identify any opportunities you see for increasing information flow across and between disciplines to deliver high-quality care to this patient.

**Figure 6:**
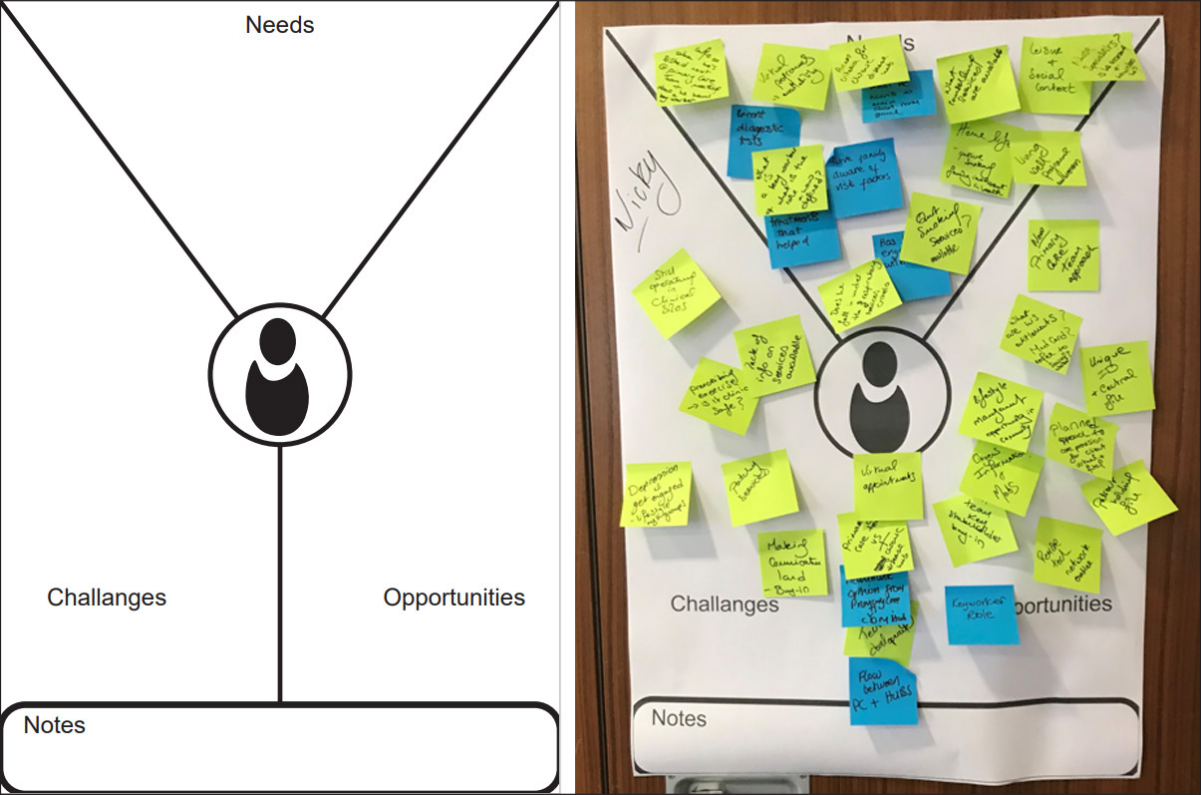
Persona Needs Finding template (Blank: Left) with in-process affinity diagramming (Right).

As with Part 1 of the workshop, as experiences were shared, they were written on sticky notes and displayed on the ‘Persona Needs Finding template’ (Figure 6) in a process of affinity diagramming.

#### Interim analysis phase

An interim analysis phase was conducted by the design and facilitation team to organise identified themes and formulate a current position understanding from workshop 1. Workshop data were analysed through a grounded theory approach. This was a process of data management and coding [33]. Firstly, data management involved ‘cleaning’ [34] or organising the data and transcription (of all sticky notes on posters). This process was commenced during the workshop where data was organised with participants into broad themes. A deeper thematic analysis and synthesis occurred post-workshop through the coding of all sticky notes on posters. Coding is the “sensemaking” stage where analytic questions will be asked of the data, categorising segments with a short name (a code) and using these codes to develop a deep understanding of what is happening [35,36]. A combination of manual coding with the CAQDAS software NVivo™ was used to code the data. Approx. 450 sticky notes were coded into 50 themes in the first pass of coding. The second pass at coding synthesised these into 3 main themes with 12 sub-themes. Analysis and themes from co-design workshops were initially reported back to participants in workshop 2, with complete workshop findings presented at HSPC group presentations. Analysis and identified themes were key outputs from workshop 1.

#### Co-Design Workshop 2: The Future Position

The objective of co-design workshop 2 was to envision and develop a ‘future position’ for improved information needs, to enable person-centred integrated care. The findings in Workshop 1 created a platform to build the Workshop 2 agenda concentrating on moving into a solution-focused space with identified priorities and workable solutions for service development. The co-design activity was conducted in an active workshop-style setting, a large hotel function area was hired with moveable furniture to allow for flexibility and movement, and large wall spaces to visualise and share ongoing work. This was a half-day event, facilitated in person. Table 3. outlines the agenda for workshop 2. Similar to workshop 1, in the planning stage of the workshop, prototyping of workshop assets, ‘trial runs’ and iterative revision of the process occurred. Reflections and learning from the first workshop were implemented into the second workshop.

**Table 3:** Agenda for Workshop 2.


INFORMATION NEEDS FOR INTEGRATED WORKING – WORKSHOP 2 AGENDA	

**8:45 am**	Tea/coffee and networking.

**9:15 am**	Welcome, introductions and looking to the future

**9:30 am**	Workshop 1 – Overview of Findings

**9:45 am**	Analysis and Themes of Workshop 1

**10:00 am**	Breakout 1 – How will my working day be better in the Network?

**10:30 am**	Coffee break

**10:50 am**	Breakout 2 – Identifying solutions and next steps

**12:00 pm**	Closing remarks

**12:15 pm**	Lunch


#### Workshop Format

The introduction again outlined the importance of the co-design approach highlighting that participant input was essential to the development of new ways of working in the Network. Following the introduction, an overview of findings from workshop 1 was presented, serving as a reminder of the process and showing the range of responses. Analysis and themes of workshop 1 were then presented followed by an overview and objectives of workshop 2.

workshop 2 followed a similar format to Workshop 1. Participants (N = 18) were grouped into 3 breakout groups of 5 to 7 guided by one of the 6 facilitators. Groups were selected to ensure diversity in an interdisciplinary range of healthcare providers. The workshop was designed to be open and discursive; to collect as many insights, views, ideas, and narratives of services as possible, building on and exploring the ideas of others. As ideas were shared, they were written on sticky notes and displayed in a process of affinity diagramming. In contrast to Workshop 1, one canvas was used to facilitate the entire workshop. This was in the format of a ‘Near, Mid and Far’ timeline canvas (outlined in Table 4).

**Table 4:** Format for ‘Near, Mid and Far’ timeline canvas used in workshop 2.


	NEAR (6–12 MONTHS)	MID (1–3 YEARS)	FAR (3 YEARS+)

**Theme 1** **Increased awareness of services in the Network (Who What Why Where and When)**			

**Theme 2** **Integrated Team working within and beyond the Network**			

**Theme 3** **Service Development and delivering on common goals**			


#### Co-Design Workshop 2: Breakout 1

Breakout 1 was centred on the following question: How will my working day be better in the Network? Each group shared ideas based on “I would like to have” or “if I had” statements in relation to the following overarching themes that were identified within Workshop 1:

- Theme 1: Increased awareness of services within the Network.
- Theme 2: Integrated team working within and beyond the Network.
- Theme 3: Service developments and delivering on common goals in the Network.

Each group worked across the canvas envisioning the future position over periods: Near = 3–6 months, mid = 1–2 years, Far = 3–5 years.

#### Co-Design Workshop 2 Breakout 2 – Identifying solutions and next steps

Continuing with the canvas and the statements identified from breakout 1 group focused on 2 actions in breakout workshop 2. Groups were asked to identify challenges and to create solutions to these challenges led by the questions:

1. What are the challenges to implementation?
2. What are the potential solutions to those challenges?

#### Analysis of Workshop 2

**Figure 7:**
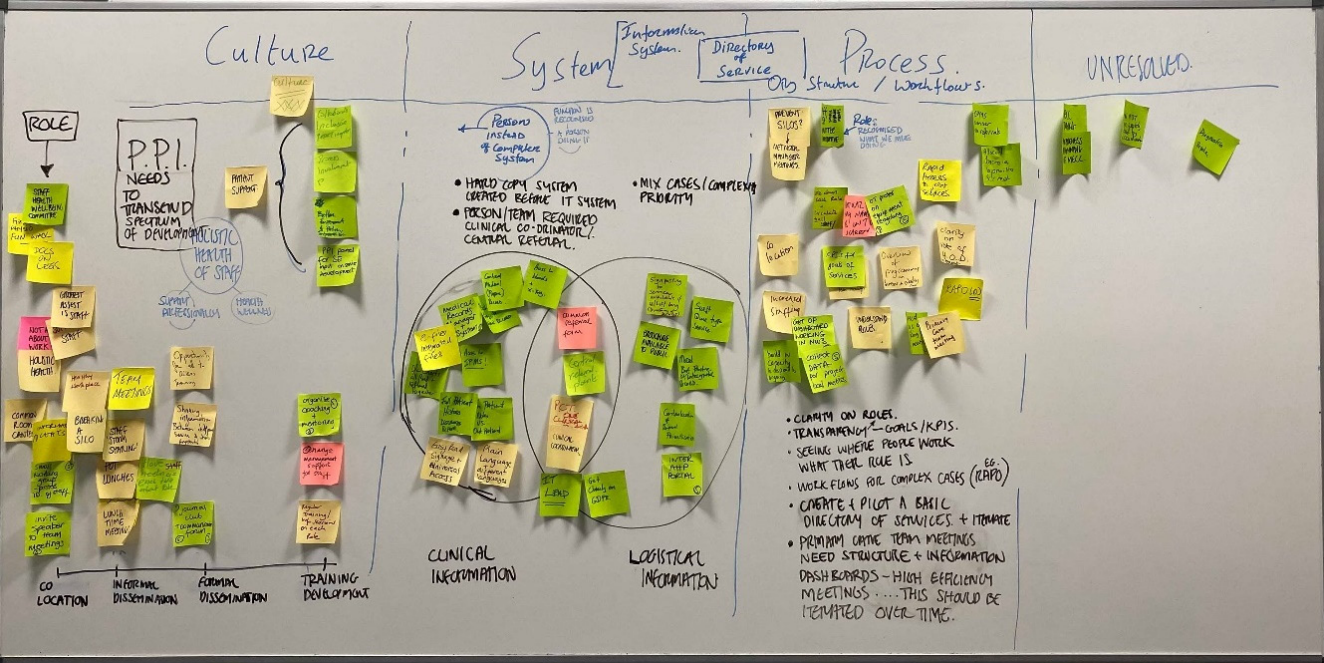
Workshop 2 process of manual coding into themes on a whiteboard.

The analysis of the outputs of workshop 2 was conducted through a process of manual coding (Figure 7) of the insights into organising themes and visual analysis and sense-making of the resultant insights and themes [37,38]. This was achieved with two facilitators coding the insights. To start the analysis, firstly, the insights gathered on the workshop canvas were grouped into observations, challenges, and solutions and these were spaced by timeframe from immediate and near-term through to perfect future position (long term). Notes carrying solutions were identified either by the colour of the note or a marking applied to the note by the participants in the workshop. With the solutions and insight viewed in this manner, organising structures were developed.

Identified from the first pass (independent observations) the following overarching observations were:

- The importance of building social connections within the workspace to facilitate enhanced communication.
- The ongoing effectiveness of staff to be supported with attention to well-being and staff training.
- Information system needs and improvement of systems support in current needs.
- Importance of establishing common frames of reference.
- Requirement for clarity around organisational structures and potential to restructure around network delivery.
- Value and challenge around integrated team working.

Combining these independent insights resulted in the proposal of three key overarching but interconnected themes which served as information outputs from the workshops:

1. Culture
2. Systems (Information Systems)
3. Processes (organisational structures and workflows)

These themes including complete workshop findings were presented at HSPC group presentations and reported back to participants.

## Discussion: 4 Key learning points on Co-designing with Integrated Care Teams

A specific objective of this paper is to reflect on the experience of co-designing used within integrated care to inform the ‘how to do’ of future practice. The following is a reflection synthesised into 4 key points. This was conducted by the design and facilitation team using the Gibbs reflective cycle.

1. **Preparation, Pre Planning and Planning for post-workshop analysis are key**
  • Pre-planning and tailoring of workshops prior to running and implementation are most important. ‘Trial runs’ of the process before workshops and prototyping of workshop assets to see how they may be interpreted by participants were key. In the planning stage, workshop assets, workflow, and timings should be revised to anticipate and mitigate potential adverse responses. This can help in mitigating potential challenges e.g., power and dynamics of participants, energy, and diversity of voice within participants.
  • Plan and prepare to make the best use of time. Professionals with high workloads need to feel workshops are fully considered and that their time is respected and well spent.
  • It is also important to allow for flexibility, iteration, and refinement as co-design workshops progress. Also, to allow for an improvement/learning cycle between workshops, in this instance, the second workshop was refined based on learning from the first.
  • Plan for analysis and synthesis. This can be a labour-intensive and time-consuming activity to ensure rigour, especially manual coding. Factor in sufficient time for this activity and resources when applying for funding to conduct co-design.
2. **Ensure Simplicity in Format**
  The process of co-designing in groups benefits from being visual [39,40] and designing simple inclusive formats with easy-to-interpret assets. From the perspective of facilitation, simplification of assets to record workshops e.g., the use of simplified posters and specifically created personas were key for engagement. This allows for time efficiency in facilitation and elicits quick and diverse inputs from participants.
3. **Interdisciplinary Co-design and Co-facilitation of Workshops**
  • The design and facilitation team in this instance consisted of design practitioners together with subject matter experts, healthcare managers and clinicians. Having an interdisciplinary team offers benefits both in terms of designing appropriate workshops and assets and the ability for healthcare practitioners to replicate the activity for future studies, independent of the support of the design practitioners.
  • Input of integrated care team managers is critical to ensure that findings from co-design processes are honoured and implemented where possible. This supports staff in their transitioning to new integrated team structures and allows an understanding of their value within the service they provide while simultaneously delivering on the quadruple aim of Sláintecare.
4. **Co-designing can enhance communication for service improvement**
  • Facilitators felt that the co-design workshops helped in enhancing interdisciplinary communication. The act of getting a diversity of healthcare staff including HSCPs and their Public Health Nursing and GP colleagues in a room together to discuss integrated care was considered powerful and important in itself.
  • The design and facilitation team felt that co-design in this instance provided a means for the workforce to identify and develop different ways of working together, to sustain this practice and improve. The workshops were seen to identify better ways of working to support integrated care, identifying a continuum of activities to build integrated care capacity.

## Conclusion

This paper aimed to inform future practice in the understanding of the development, use and facilitation of co-design with integrated care teams. Conducted through a process of quality improvement, we sought to establish a shared understanding of information needs among a newly formed integrated care team. We developed and used co-design methods to understand the ‘current and future position’ of service information needs from the team perspective to support good practice in integrated care provision.

The workshops offered a wide range of service information needs. Culture Building, System Needs, and Processes were identified as key themes in future information needs for healthcare staff working in CHNs. Offering a reflection of methods used, we conclude with 4 key learning points on co-designing with Integrated Care Teams. 1. Ensure simplicity in format. 2. Interdisciplinary co-design and co-facilitation of workshops are beneficial. 3. Planning and preparation of workshops are key. Finally, 4. From this work, we conclude that co-designing can enhance communication for service improvement.

Future co-design work will now be progressed to help develop these findings and evolve best practices in integrated care service improvement. This future work will focus on the information needs of both service staff and users ensuring equality and diversity of voice of all participants.
